# Early-life stress leads to impaired spatial learning and memory in middle-aged ApoE4-TR mice

**DOI:** 10.1186/s13024-016-0107-2

**Published:** 2016-07-12

**Authors:** Lan-yan Lin, Jing Zhang, Xiao-man Dai, Nai-an Xiao, Xi-lin Wu, Zhen Wei, Wen-ting Fang, Yuan-gui Zhu, Xiao-chun Chen

**Affiliations:** Department of Neurology and Geriatrics, Fujian Institute of Geriatrics, Affiliated Union Hospital of Fujian Medical University, 29 Xinquan Road, Fuzhou, 350001 China; Key Laboratory of Brain Aging and Neurodegenerative Diseases, Fujian Key Laboratory of Molecular Neurology, Fujian Medical University, 29 Xinquan Road, Fuzhou, 350001 China; Department of Neurology, Affiliated Union Hospital of Fujian Medical University, 29 Xinquan Road, Fuzhou, Fujian 350001 People’s Republic of China

**Keywords:** CUMS, Major depressive disorder, Apolipoprotein E genotype, Cognitive impairment, Reelin

## Abstract

**Background:**

Apolipoprotein E (ApoE) is a major lipid carrier that supports lipid transport and injury repair in the brain. The *APOE* ε4 allele is associated with depression, mild cognitive impairment (MCI) and dementia; however, the precise molecular mechanism through which ApoE4 influences the risk of disease development remains unknown. To address this gap in knowledge, we investigated the potential effects of chronic unpredictable mild stress (CUMS) on ApoE3 and ApoE4 target replacement (ApoE3-TR and ApoE4-TR) mice.

**Results:**

All ApoE-TR mice exposed to CUMS at 3 months old recovered from a depression-like state by the age of 12 months. Of note, ApoE4-TR mice, unlike age-matched ApoE3-TR mice, displayed impaired spatial cognitive abilities, loss of GABAergic neurons, decreased expression of Reelin, PSD95, SYN and Fyn, and reduced phosphorylation of NMDAR2B and CREB.

**Conclusion:**

These results suggest that early-life stress may mediate cognitive impairment in middle-age ApoE4-TR mice through sustained reduction of GABAergic neurons and Reelin expression, which might further diminish the activation of the Fyn/NMDAR2B signaling pathway.

## Background

Apolipoprotein E (ApoE) is a major lipid carrier that plays an important role in maintaining lipid homeostasis, both in the periphery and the brain, and in various physiological processes, including central nervous system development, nerve regeneration and repair, as well as learning and memory [1, 2]. The human *APOE* gene has three polymorphic alleles, namely *APOE2*, *APOE3*, and *APOE4*. Although various genetic backgrounds and life experiences may cause differences in adaptability and response capability of individual brains to stressful events [3], ApoE4 has been documented to be an age-dependent and a gene-dose-effect risk factor for late-onset familial and sporadic Alzheimer’s disease (AD) [4–6] and psychiatric disorders, such as depression [7].

Recent studies have reported that early-life symptoms of depression can increase the risk of cognitive impairment in old age [8–10], and that adverse events in childhood have a more severe effect on the depressive symptoms present in older-age ApoE4 carriers, as compared to ApoE4 non-carriers [11]. Emerging clinical evidences indicate that compared with ApoE4 non-carriers, depression patients carrying an *APOE4* allele have significantly-reduced hippocampal volume [12]; and patients with Alzheimer’s disease, who have a life-time history of Major Depression, have increased hippocampal plaques and tangles [8]. In addition, a prospective study of 142 twins found that patients with dementia, tardive depression, and the *APOE4* allele exhibit an increased risk for AD pathogenesis [13]. Other prospective studies also confirm a close association among depression, *APOE* genotype and mild cognitive impairment [14, 15]. These findings suggest that there is a positive correlation between depressive symptoms and cognitive decline in people carrying one or two *APOE4* alleles [16]. However, the potential mechanisms underlying the relationships among depression, *APOE* genotype, and mild cognitive impairment remain largely unknown.

Previous studies have verified that patients with schizophrenia or bipolar disorder/manic depression suffer a great loss of GABAergic neurons in their prefrontal cortex [17]. GABAergic neurons can secrete the glycoprotein Reelin, which plays an important role in regulating synaptic plasticity [18]. In the brain, Reelin mainly binds to two major lipoprotein receptors on the cell membrane, apolipoprotein E receptors 2 (ApoER2) and very-low-density lipoprotein receptor (VLDLR) [19]. Binding of Reelin to the receptors induces feed-forward activation of DAB1, an adaptor protein that interacts with NPxY motifs in both receptor tails [20]. The clustering of DAB1 activates SRC family tyrosine kinases (SFKs), then Reelin-activated SFKs phosphorylate the NMDAR on NR2 subunits, resulting in the potentiation of NMDAR-mediated Ca^2+^ influx. Elevated intracellular Ca^2+^ can activate the transcription factor cyclic AMP response element binding protein (CREB), thereby potentially initiating the expression of genes that are important for synaptic plasticity, neurite growth and dendritic spine development [20–22]. Deficits in Reelin have been documented to be closely associated with mental illness, such as schizophrenia and depression in human subjects [23, 24]; symptoms of mental illness and cognitive impairment in Reelin-knockout mice have also been reported [25].

Given existing studies are largely population-based and the observed phenomena have not been confirmed in ApoE-TR mouse models, we sought to determine whether early-life depression in ApoE4 target replacement (ApoE-TR) mice impairs cognitive function through a loss of GABAergic neurons and perturbations in the Reelin-ApoER2 signaling pathway. To test this hypothesis in the current study, we applied a 6-week chronic unpredictable mild stress (CUMS) procedure to 3-month-old ApoE-TR mice, thereby generating a reliable depression model that mimics a human depressive state [26]. We found that ApoE4-TR mice that underwent the early CUMS procedure displayed cognitive impairment at 12-months-old and exhibited decreased GABAergic neurons in the prefrontal cortex and the dentate gyrus (DG) of the hippocampus. Furthermore, the expression levels of Reelin and its down-stream signaling molecules (Src family tyrosine kinases Fyn and NMDAR receptor subunits 2B) were significantly reduced. Taken together, our novel findings demonstrate that early CUMS may lead to impaired cognitive function via the Reelin-ApoER2-Fyn signaling pathway in middle-aged ApoE4-TR mice.

## Methods

### Animals and experimental protocol

Human ApoE-TR homozygous mice of the C57BL/6 J background were obtained from the Taconic (www.taconic.com), in which the expression of the human ApoE2, ApoE3, or ApoE4 is controlled by the mouse ApoE promoter [27]. The colony was maintained by homozygous breeding. Young male ApoE3-TR mice (aged 10 weeks, *n* = 60) and age-matched ApoE4-TR mice (*n* = 60) were respectively randomized into two groups: those maintained under chronic stress conditions, the CUMS group (*n* = 30), and those maintained under normal conditions, the control group (*n* = 30). All animals were housed in standard plastic cages (4-5 mice per cage) with wood chips for bedding at 22 ± 1 °C under a relative humidity of 55 ± 5 % and a twelve-hour light/dark cycle (light from 6:00 a.m. to 6:00 p.m.). They were allowed free access to food and water. All experimental protocols and procedures were approved by the Committee of Institutional Animal Care and Use of Fujian Medical University and closely observed the “Guide for the Care and Use of Laboratory Animals” by the U.S. National Institutes of Health (NIH Publications No. 80-23, revised in 1996).

**Fig. 1:**
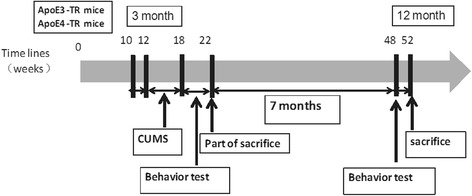
The time schedule for experimental procedures in ApoE-TR mice. During weeks 10–12, all ApoE3-TR mice (*n* = 60) and ApoE4-TR mice (*n* = 60) received sucrose preference training and environmental adaptation. A group of ApoE3-TR and ApoE4-TR mice underwent the CUMS procedure during weeks 12–18; all mice underwent behavior testing during weeks 18–22. About 50 % of ApoE3-TR and ApoE4-TR mice were sacrificed during week 22 with the remaining mice undergoing behavior testing during weeks 48–52 prior to sacrifice

All the animals received sucrose preference training and environmental adaptation for 2 weeks; after which the CUMS groups underwent the CUMS procedure for 6 weeks. After the intervention, the animals received behavioral tests for a period of 4 weeks. At the end of the behavioral tests, some of the mice were sacrificed as samples; the rest were normally bred to 12 months and underwent the behavioral tests again before sacrifice for further experiments. The time schedule is provided in Fig. 1.

### Chronic Unpredictable Mild Stress (CUMS) procedure

The stress procedure was modified from a previous description by Willner et al. [26, 28]. In brief, for the chronic stress group, each mouse was kept in isolation in a single cage and underwent a variety of mild stressors: white noise for 12 h, cage tilting for 24 h, swimming in cold water (18 °C) for 5 min, food deprivation for 12 h, water deprivation for 12 h, wet bedding for overnight, and strobe lights for 24 h, restrictions in a small tube (10×5×5 cm) for 2 h. The stress procedure continued for 6 weeks prior to behavioral tests; the control animals were kept in groups (five mice per cage) and given ordinary daily care.

### Sucrose solution consumption test

As previously described [29, 30], the sucrose preference training began prior to the stress procedure and the sucrose solution consumption test was conducted once a week during the stress procedure. The detailed test protocol was as follows: after a 12-h period of water and food deprivation, the sucrose preference test was performed from 10:00 am to 12:00 am; the animals were allowed free access to two bottles containing water and 1 % sucrose solution respectively; two hours later, the volumes of water and sucrose solution consumed were measured. The amount of sucrose solution ingested as a percentage of the total liquid was interpreted as the parameter of hedonic behavior.

### Behavioral testing

During the behavioral test, the CUMS procedure was continued without food and water deprivation. All of the tests were conducted in a test room with relatively dim lighting (±20 lux) from 8:00 a.m. -11:30 a.m. Mice were given 30 min to adapt to the environment before the test began and returned to their original location immediately after the experiment.

#### Elevated plus maze test

The elevated plus-shaped maze test was employed as described by Pellow [31, 32]. The maze apparatus (50 cm off the ground) consisted of two open arms (50 × 5 cm; with ledges, 0.5 × 0.5 cm) and two opposite closed arms (50 × 5 cm; with walls, 38 cm high), forming a middle square area (5 × 5 cm), which allows the animals to pass through the arms freely. Mice were individually placed in the middle square area and allowed free exploration for 10 min. Their behaviors were recorded by a video tracking system (Super Maze V2.0, XinRuan, Shanghai, China) and the following variables were analyzed: the frequency and distance animals traveled into the open arms, closed arms and middle square area. After each behavioral test, the apparatus was cleaned with 75 % alcohol-water to remove odors.

#### Open field test

The open field test (OFT) was designed in accordance with the procedures described by Heimrich et al. [33]. Each mouse was placed in the middle of an open box (50× 50 × 50 cm) and the area was divided into 9 squares of 16.67 cm × 16.67 cm with painted white lines. Activity was recorded by a digital camcorder (Sony, Japan) and analyzed using Top Scan software (Super Maze V2.0 XinRuan Information Technology Co. Ltd, Shanghai, China). Indicators included horizontal movement (the number of crossings as assessed by the grids marked on the bottom of the box) and vertical movement (the times of rearing) during the 10-min test. To eliminate the interference of animal odor, the box was cleaned with 75 % alcohol between testing sessions.

#### Tail suspension test

Immediately after the OFT, the tail suspension test (TST) was performed as described by Crowley et al. [34]. In brief, we trussed up the mouse tail with a piece of adhesive tape and fixed it on a hook (about 2 cm away from tip) and suspended the mouse approximately 28 ± 2 cm off the floor. Then we recorded the duration of behavioral immobility of each mouse within a period of 6 min with an automated TST device (Super Maze V2.0, XinRuan, Shanghai, China). The experiment was conducted according to the following parameters: threshold 1 = 7, gain = 16 times, constant = 0.25, resolution = 200 ms. Data were analyzed by technicians who were trained but blind to the experiment protocol.

#### Morris maze test

The water maze apparatus and procedures have been described in our previous research [35]. Briefly, the dark stainless steel pool was 1.2 m in diameter and 0.5 m high with a blank nontoxic plastic bottom. A round platform made of transparent plexiglas (7 cm in diameter) was placed in the center of the southeast corner. Before tests, the circular pool was filled with water to a depth of 35 cm,approximately 2 cm higher than the height of the platform. The water was rendered opaque by pouring 1500 ml of milk into the pool and the temperature of the water and the test room was set at 22 ± 2 °C during the test.

During cognitive spatial ability testing, each animal took four trials daily for 5 consecutive days. Each trial was started from a different location and lasted for 60 s, with the mouse facing the wall of the pool when placed into the water. The quadrant from which the mouse was placed into the water in each trial was varied according to semi-random sequence distribution decisions by Vorhees & Williams [36]. On locating the platform, the mouse was left there for 15 s before the next trial. If the animal failed to locate the platform within 60 s, it was guided to the platform and allowed to stay there for 15 s. The latency and the travel orbit of reaching the platform were recorded by the tracking system. On the sixth day, the learning memory test was conducted with the platform removed. Each animal was given 60 s to explore the pool. Mouse performance was recorded by Smart 2.0 video-tracking software (PanLab, Barcelona, Spain).

### Tissue preparation

Mice were deeply anesthetized with 10 % chloral hydrate (3 ml/kg) by intraperitoneal injection and perfused via the left ventricle with ice-cold 0.1 M phosphate-buffered saline (PBS) (25 ml per mouse). Their brains were rapidly removed from the skull and dissected on ice. The prefrontal cortex and hippocampus of some of the harvested brains were isolated and dipped into liquid nitrogen and stored at -80 °C. The remaining regions were fixed in 4 % paraformaldehyde at 4 °C for 48 h and dehydrated twice with 30 % sucrose solution at 4 °C for 24 h each time. The fixed brains were cut into serial sections (30 um thick) with a freezing microtome (CM1850, Leica, Germany) and the sections were picked up in antifreeze liquid (30 % glycerol, 30 % ethylene glycol, 40 % 0.1 M PBS) and stored at -20 °C until use.

### Western blot analysis

Tissues were dissected from 3-month-old and 12-month-old mice and homogenized in a lysis buffer (30 mM Tris-HCL, 2 mM Na3VO4, 50 mM NaF, 10 mM Na4P2O7, 1 % Triton X-100 and 1 % protease inhibitor cocktail at pH 7.4). Then, the supernatants were collected by centrifuging at 16,000 g at 4 °C for 25 min. Protein concentration was determined with the Bradford assay kit (Bio-Rad, Hercules, CA, USA) and adjusted to 2.0 mg/mL with lysis buffer and 6× sample buffer (125 mM Tris, pH 6.8, 0.006 % bromophenol blue, 130 mM dithiothreitol, 10 % sodium dodecyl sulfate and 10 % glycerol). Equal amounts of proteins were heated at 100 °C for 5 min. The total protein lysates were separated by 10–12 % sodium dodecyl sulfate–polyacrylamide gel electrophoresis (SDS-PAGE) and transferred to polyvinylidene difluoride (PVDF) membranes (0.25 micrometer) overnight using wet transfer equipment at 90 mA (Bio-Rad Laboratories, Hercules, CA, USA). The membranes were blocked in 5 % bovine serum albumin (BSA) in Tris-buffered saline Tween-20 (TBST, pH 7.6, containing 10 mM Tris, 150 mM NaCl, and 0.1 % Tween-20) at room temperature (RT) for one hour, followed by incubation with primary antibodies diluted in 2.5 % BSA/TBST at 4 °C overnight. After incubation, the membranes were washed in TBST three times (10 min per time), and further incubated off light at RT for one hour with infrared dye-labeled fluorescence secondary antibodies diluted in TBS (IRD 800cw, goat-rabbit C40325-02, goat-mouse C40213-01, 1:10000; LI-COR, USA). Then, the membranes were washed three to four times in TBST (10 min per time) before fluorescence detection with Odyssey Sa color infrared laser imaging system (LI-COR, USA) and densitometry analysis with NIH Image J software. The antibodies used were as follows: mouse anti-tubulin (Sigma,1:50000), rabbit anti-PSD-95 (postsynaptic density protein 95) (Millipore,1:2000), mouse anti-Reelin (Milipore, 1:500), mouse anti-APOE (Santa-cruz, 1:500), and rabbit anti-NMDAR2B, rabbit anti-phosphorylation-NMDAR2B, rabbit anti-APOER2, mouse anti-Fyn (Abcam, 1:1000, respectively), mouse anti-SYN (synaptophysin) (Millipore 1:10000), rabbit anti-phosphorylated CREB (Millipore 1:500), rabbit anti-CREB (Abcam 1:1000).

### Immunohistochemistry

For immunodetection of GABAergic neurons and Reelin, the sections were washed with Tris-buffered saline TBS for 6×10 min and immersed in 3 % H2O2/TBS to inactivate endogenous peroxidase in the dark for 20 min. Next, they were washed with TBS (3×10 min) and blocked with TBS containing 0.3 % Triton X-100, 0.25 % bovine serum albumin (BSA), and 5 % goat serum (GS) at RT for 2 h. Then, they were incubated overnight at 4 °C with primary antibodies (anti-GABA, rabbit, 1 : 8000, Sigma or anti-Reelin, mouse, 1:2000, Millipore) in TBS containing 0.25 % BSA, 2 % normal goat serum, and 0.3 % Triton X-100. After the incubation, they were washed with TBST for 6×10 min, and further incubated at RT for 90 min with biotinylated secondary antibodies (at 1: 600, Vector Laboratories, Burlingame, CA, USA). After further washes with TBST (6×10 min), they were incubated in Vector Elite avidin–peroxidase dilution (at 1:200) at RT for 60 min. Subsequently, the above-treated sections were serially washed in TBST (3×5 min) and in 0.175 M sodium acetate solution (3×5 min) before the staining was revealed with diaminobenzidine (DAB) and H2O2 diluted in 0.175 M sodium acetate at RT for 10 min and terminated with 0.175 M sodium acetate promptly. They were then mounted on glass slides, which had been coated with poly-lysine and air dried at RT overnight. Finally, they were dehydrated with graded alcohol and rendered transparent by xylene liquid, and coverslipped with a permanent mounting medium (Vector Laboratories, USA).

The stained sections were observed under a microscope (OlympusBX-51, Olympus, Japan). Image acquisition was performed with Image-Pro Express 5.1 image analysis software. For quantitative analysis, we randomly selected 4–5 mice from each group and measured 3–5 consecutive sections of each mouse. The prefrontal cortex and dentate gyrus (DG) region of the hippocampus were selected as regions of interest (ROI) and the identical area within the measuring frame in a 10X objective lens was labeled. The number of positively-stained neurons in the frame was counted by 100X magnification. The clear brown cellular boundaries were considered positive, although positive cells outside the frame were rejected. “Cells” that were lightly stained or had irregular shapes were excluded from quantification. Then the mean value for each mouse was calculated.

### Immunofluorescence

For immunofluorescence staining of the Glutamatergic neurons, the sections were washed with Tris-buffered saline (TBS) for 6×10 min, next blocked with TBS containing 0.3 % Triton X-100, 0.25 % bovine serum albumin (BSA), and 5 % donkey serum (DS) at RT for 2 h. Then, they were incubated overnight at 4 °C with primary antibodies (rabbit anti-VGluT1, Abcam 1:1000) in TBS containing 0.25 % BSA, 2 % normal donkey serum, and 0.3 % Triton X-100. After the incubation, they were washed with TBST for 6×10 min, and further incubated at RT for 90 min with fluorescent secondary antibodies (Alexa Fluor 488-conjugated donkey anti-rabbit IgG, Invitrogen, 1: 2000). After further washes with TBST (6×10 min), they were then mounted on glass slides coated with poly-lysine. Then the prefrontal cortex and dentate gyrus (DG) region of the hippocampus were selected as regions of interest (ROI) and taken pictures by the Confocal Microscopy (Zeiss, 780). The Mean fluorescence intensity was analyzed in the all groups.

### Real-time reverse transcription polymerase chain reaction

Total RNA was extracted from the prefrontal cortex and hippocampus using TriPure Isolation reagent (Roche, Mannheim, Germany) according to the manufacturer’s protocol and was reverse transcribed using the Transcriptor First Strand cDNA synthesis kit (Ferments, Canada). Polymerase chain reaction (PCR) was performed with Fast Start Universal SYBR Green Master (Roche), and fluorescence was measured using the Step-One Plus realtime PCR system (Life Technologies Applied Biosystems, Grand Island, NY). The following primer sets were used: Reelin (NM_011261, sense 5-GGACTAAGAATGCTTATTTCC-3 and anti-sense 5- GGAAGTAGAATTCATCCATCAG -3) and GAPDH (NM_008084, sense 5-CAGTGGCAAAGTGGAGATTGTTG -3 and antisense 5- CTCGCTCCTGGAAGATGGTGAT -3). Each reaction was run in triplicate. The efficiency of all experiments fell between 95 and 105 %, and all gene measures displayed normal melt curves. Fold changes were calculated by 2^−Δ(ΔCt)^ [ΔCt = Ct (target gene) – Ct (GAPDH); Δ(ΔCt) = ΔCt (experimental groups) − mean ΔCt (3-month control-E3 groups)].

### Statistical analysis

Data were analyzed with SPSS 13.0 statistical software and quantitative data were expressed as mean ± SEM. Data sets were first tested for normal distribution and then compared using,one-factor and two-factor ANOVA. Statistical significance was set at *p* < 0.05.

## Results

### CUMS procedure successfully induces depression-like behaviors in 3-month-old ApoE-TR mice

**Fig. 2:**
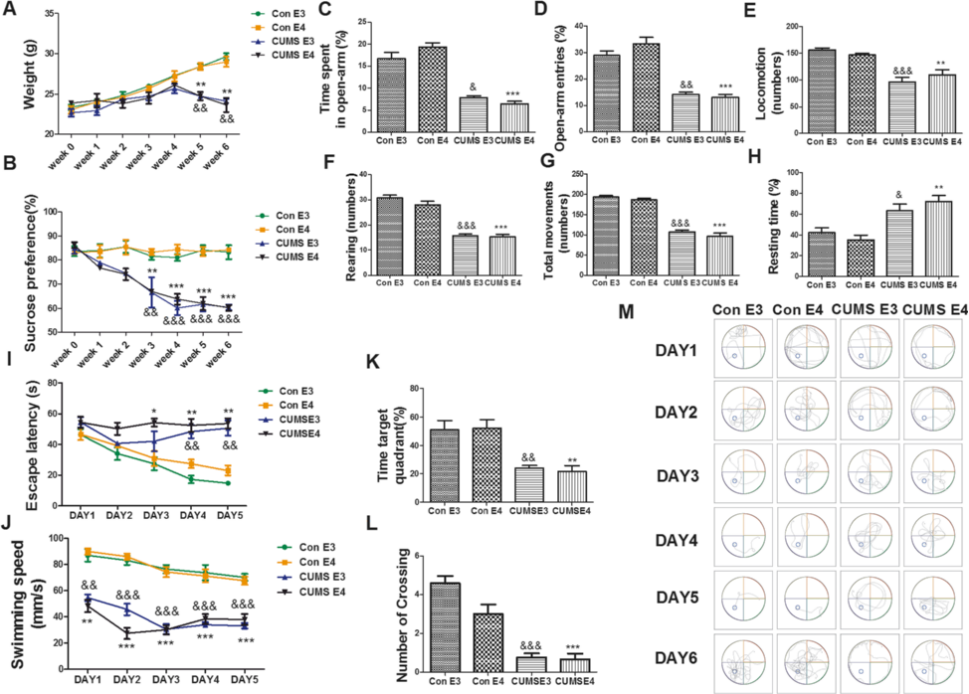
CUMS successfully induced depression-like behaviors in 3-month-old ApoE-TR mice. **a**-**b** Weight change and the percentage of sucrose preference in 3 M ApoE-TR mice during 6 weeks of CUMS. **c**-**d** The percentage of time spent and number of entries into the open arms for 3 M ApoE-TR mice in the elevated plus maze test. **e**-**g** The extent of locomotion (Horizontal), rearing (Vertical) and total movement (Horizontal + Vertical) for 3 M ApoE-TR mice in the open field test. **h** The percentage of resting time for 3 M ApoE-TR mice during the 6 min tail suspension test. **i** Escape latency in 3 M ApoE-TR mice for the first 5 days during the Morris maze test. **j** The swimming speed of 3 M ApoE-TR mice for the 5 days during the Morris maze test. **k**-**l**) The percentage of time spent in target quadrant and the number of times crossing the former platform position during the last day of 3 M ApoE-TR mice in the Morris maze test. **m** Representative swimming tracks in the Morris water maze for days 1–5 of training and day 6 of probe trial. *N* = 12 per group, expressed as mean ± SEM. The Figure **a**, **b**, **i**, **j** used the two-factor ANOVA of statistical methods, and the Figure **c**-**h**, **k**, **l** used the one-factor ANOVA of statistical methods. & *p* < 0.05, && *p* < 0.01, &&& *p* < 0.001, CUMS E3 vs. Con E3. *p < 0.05, **p < 0.01, ***p < 0.001, CUMS E4 vs. Con E4

To assess the impact of the CUMS procedure, we evaluated the weight, sucrose consumption and behavior of ApoE3/4-TR mice undergoing CUMS compared to controls. While the weights of mice in the control groups steadily increased during weeks 12–18, the weights of mice undergoing CUMS leveled off, with a significant difference between the control groups and the ApoE4-TR mice observed in the fifth week (for ApoE3, 24.73 ± 0.28 vs. 28.41 ± 0.35, *p* < 0.01; for ApoE4, 24.74 ± 0.52 vs. 28.41 ± 0.43, *p* < 0.01) (Fig. 2a). Sucrose consumption preference in the CUMS groups gradually decreased, with the difference between the CUMS groups and the controls becoming significant in the third week (for ApoE3, 61.63 % ±2.95 % vs 84.12 % ± 2.08 %, *p* < 0.01; for ApoE4, 61.94 % ±2.73 % vs. 83.44 % ±1.89 %, *p* < 0.01) and greatly intensifying in the sixth week (for ApoE3, 60.41 % ±1.15 % vs. 83.22 % ±2.86 %, *p* < 0.001; for ApoE4, 60.17 % ±1.20 % vs. 84.25 % ± 0.57 %, *p* < 0.001) (Fig. 2b).

To assess whether the CUMS groups exhibit depression-like behavior, an elevated plus maze, open field test and tail suspension test were used. Compared with the control groups, the CUMS groups spent significantly less time in the open arms of the elevated plus maze (for ApoE3, 7.87 % ± 0.45 % vs. 16.74 % ± 1.42 %, *p* < 0.05; for ApoE4, 6.457 % ± 0.63 % vs. 19.35 % ± 0.93 %, *p* < 0.001) (Fig. 2c), and entered less frequently into the open arms after 6 weeks of CUMS intervention (for ApoE3, 14.10 % ± 0.91 % vs. 28.87 % ± 1.72 %, *p* < 0.01; for ApoE4, 13.07 % ± 1.15 % vs. 33.33 % ± 2.48 %, *p* < 0.001) (Fig. 2d). Results from the open field test demonstrated that the CUMS groups exhibited significantly reduced horizontal movement (for ApoE3, 95.83 ± 9.13 vs. 155.9 ± 4.14, *p* < 0.001; for ApoE4, 109.3 ± 10.40 vs. 146.8 ± 2.89, *p* < 0.01), vertical movement (for ApoE3, 15.77 ± 0.66 vs. 30.73 ± 1.12, *p* < 0.001; for ApoE4, 15.31 ± 1.01 vs. 28.0 ± 1.43, *p* < 0.001) and total movement (for ApoE3, 107.9 ± 3.86 vs. 193.5 ± 3.87, *p* < 0.001; for ApoE4, 97.36 ± 7.14 vs. 186.9 ± 3.16, *p* < 0.001) (Fig. 2e, f and g). Furthermore, the tail suspension test showed that the CUMS groups’ resting time, or the amount of time spent immobile, was significantly longer than the control groups, indicating a depression-like state (for ApoE3, 63.39 % ± 6.49 % vs. 42.39 % ±4.68 %, *p* < 0.05; for ApoE4, 72.33 % ± 5.66 % vs. 35.36 % ± 4.50 %, *p* < 0.01) (Fig. 2h). No significant difference was observed between the CUMS-treated ApoE3 (CUMS-E3) group and ApoE4 (CUMS-E4) groups in the above-mentioned three tests. Taken together, these data indicate that CUMS treatment successfully induces depression-like behaviors in ApoE-TR mice.

In order to further investigate changes in learning and memory, we performed the Morris water maze test, which measures hippocampus-dependent spatial navigation and reference memory. With the advancing training days, the escape latency of the control groups did gradually decrease as shown in Fig. 2i; however, the escape latency of the CUMS groups did not change regardless of training time. (for both ApoE3 and ApoE4, *p* < 0.01) (Fig. 2i). Meanwhile, the swimming speed of the CUMS groups was significantly lower than the control groups (*p* < 0.001) (Fig. 2j). In the probe trial with the platform removed, the CUMS groups spent far less time in the target quadrant (for ApoE3, 24.07 % ± 1.88 % vs. 51.13 % ± 6.47 %, *p* < 0.01; for ApoE4, 21.60 % ± 4.17 % vs. 52.36 % ± 5.83 %, *p* < 0.01) (Fig. 2k). Additionally, the number of times CUMS mice crossed the platform position was significantly less than the control groups (for ApoE3, 0.75 ± 0.21 vs. 4.58 ± 0.37, *p* < 0.001; for ApoE4, 0.66 ± 0.28 vs. 3.00 ± 0.47, *p* < 0.001) (Fig. 2l). Swimming tracks of training and probe trials were recorded for each day and each genotype (representative example, Fig. 2m). Therefore, these data indicate that the ApoE-TR mice shown a dysfunction of spatial memory in the Morris water maze after the CUMS intervention. But there was no significant difference between CUMS-E3 and CUMS-E4.

### ApoE4-TR mice recover from depression but develop cognitive impairment by 12 months old

After the initial behavioral tests, some of the ApoE-TR mice were sacrificed and brains were harvested for subsequent experiments. The remaining ApoE-TR mice were raised without additional treatment to 12 months and underwent a second round of behavioral tests to re-assess behavior and cognitive ability.

**Fig. 3:**
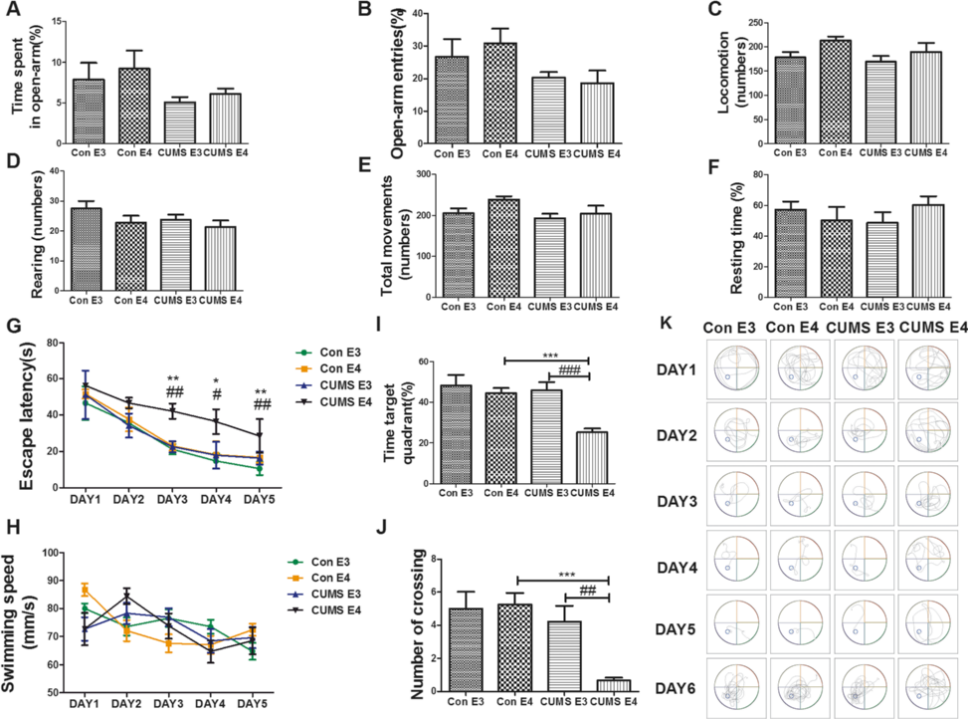
ApoE4-TR mice recovered from depression but developed cognitive impairment by 12 months old. **a**-**b** The percentage of time spent and number of entries into the open arms for 12 M ApoE-TR mice in the elevated plus maze test. **c**-**e** The extent of locomotion (Horizontal), rearing (Vertical) and total movement (Horizontal + Vertical) for 12 M ApoE-TR mice in the open field test. **f** The percentage of resting time for 12 M ApoE-TR mice during the 6 min tail suspension test. **g** Escape latency of 12 M ApoE-TR mice during the 5 days of training in the Morris maze test. **h** The swimming speed of 12 M ApoE-TR mice for the first 5 days in the Morris maze test. **i**-**j** The percentage of time spent in target quadrant and the number of platform-position crossings for 12 M ApoE-TR mice during the sixth day of the Morris maze test. **k** Representative swimming tracks in the Morris water maze for days 1–5 of training and day 6 of probe trial. *N* = 10 per group, expressed as mean ± SEM. The Figure **a**, -**f**, **i**, **j** and the Figure **g**, **h** used the one-factor ANOVA and two-factor ANOVA of statistical methods respectively.*p < 0.05 **p < 0.01, ***p < 0.001, CUMS E4 vs. Con E4. # *p* < 0.05, ##*p* < 0.01, ### *p* < 0.001, CUMS E4 vs. CUMS E3

At 12 months old, no significant difference was observed between the control and CUMS groups in the elevated plus maze test (the time percentage and the frequency percentage of entering the open arm), open field test (the horizontal movement, the vertical movement and total movement), and tail suspension test (resting time percentage) (*p* > 0.05) (Fig. 3a-b, Fig. 3c-e, Fig. 3f, respectively), suggesting that ApoE-TR mice recover from depression after CUMS intervention is terminated.

For the Morris water maze test during the 12^th^ month, the escape latency of the CUMS-E4 group was significantly greater than that of the CUMS-E3 group (*p <* 0.01 for day 3 and day 5 and *p <* 0.05 for day 4) and the control groups (for the control-E4 group, *p <* 0.01 for day 4; for the control-E3 group, *p <* 0.05 for day 4) (Fig. 3g). But the swimming speeds of all the groups did not differ noticeably from one another (Fig. 3h). The percentage of time spent in the target quadrant for the CUMS-E4 group decreased markedly in comparison with that of the CUMS-E3 group and the control group (for the CUMS-E3 group, 25.32 % ± 1.88 % vs. 45.76 % ± 4.28 %, *p* < 0.001; for the control-E4 group, 25.32 % ± 1.88 % vs. 44.50 % ± 2.51 %, *p* < 0.001) (Fig. 3i). The CUMS-E4 group crossed over the area where the platform was initially positioned markedly fewer times than the CUMS-E3 group and the control-E4 group (for the CUMS-E3 group, 0.66 ± 0.16 vs. 4.22 ± 0.95, *p* < 0.01; for the control-E4 group, 0.66 ± 0.16 vs. 5.25 ± 0.70, *p* < 0.001) (Fig. 3j). Swimming tracks during training and probe trials were recorded for each day and each genotype (representative example, Fig. 3k). No significant difference was found between the CUMS-E3 group and the control groups. Taken together, these results indicate that the CUMS-E4 mice can recover from a depressive state, but, unlike the CUMS-E3 group, their cognitive function appears to be impaired at middle-age.

### The number of GABAergic neurons, Glutamatergic neurons and expression of Reelin is decreased in the prefrontal cortex and hippocampus of 12-month-old ApoE4-TR mice that underwent early-life CUMS intervention.

**Fig. 4:**
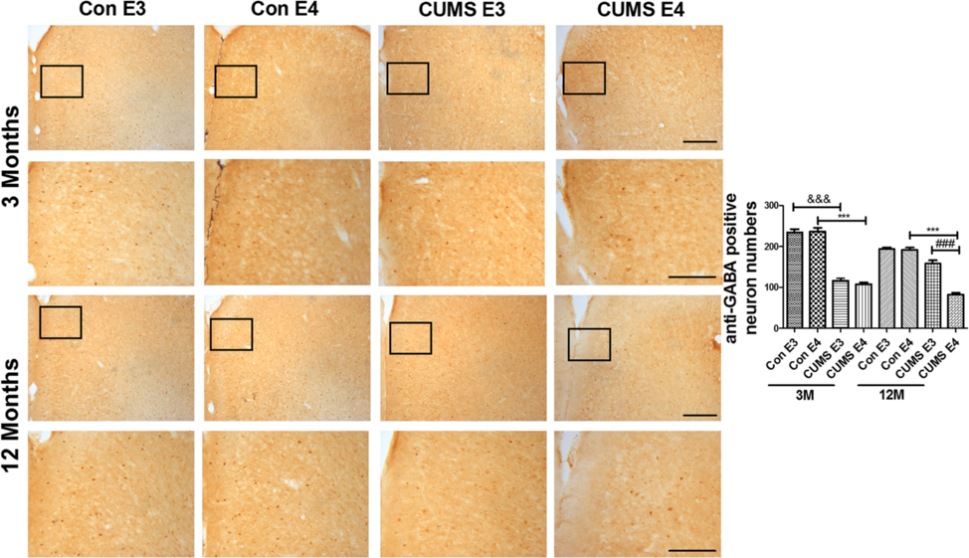
Decreased GABAergic neurons in the prefrontal cortex of 12-month-old ApoE4-TR mice that underwent early-life CUMS intervention. Immunohistochemical staining of anti-GABA in the prefrontal cortex of 3-month-old and 12-month-old ApoE-TR mice. The dark brown dots represent the anti-GABA–positive GABAergic neurons. Magnified areas indicated by boxes. *N* = 5 per group, expressed as mean ± SEM. one-factor ANOVA of statistical methods was used. &&& *p* < 0.001, ### *p* < 0.001, ***p < 0.001. Scale bars are 200 um

**Fig. 5:**
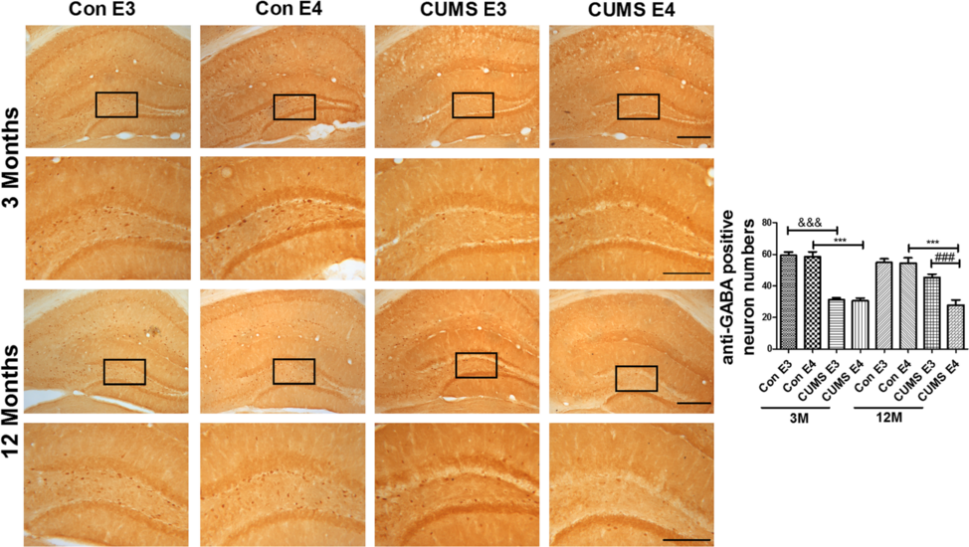
Decreased GABAergic neurons in the hippocampus of 12-month-old ApoE4-TR mice that underwent early-life CUMS intervention. Immunohistochemical staining of anti-GABA in the hippocampus of ApoE-TR mice at 3 months and 12 months. The dark brown dots represent the anti-GABA -positive GABAergic neurons. Magnified areas indicated by boxes. *N* = 6 per group, expressed as mean ± SEM. one-factor ANOVA of statistical methods was used. &&& *p* < 0.001, ### *p* < 0.001, ***p < 0.001. Scale bars are 200 um

GABAergic neurons, which are widely distributed in the prefrontal cortex and dentate gyrus (DG) region of the hippocampus, play critical roles in cognition and depression [37] and can secrete Reelin protein [38]. In order to assess the prevalence of GABAergic neurons and Reelin in controls and the CUMS group, we examined the expression of GABA and Reelin by immunohistochemistry. As shown in Fig. 4 (with brown positive staining for GABAergic neurons), the number of GABA-positive neurons in the prefrontal cortex of the 3-month-old CUMS-E3 and age-matched CUMS-E4 group was less than that of the age-matched control groups (for ApoE3, 115.5 ± 6.26 vs. 234.8 ± 7.91, *p* < 0.001; for ApoE4, 107.0 ± 4.35 vs. 237.2 ± 8.93, *p* < 0.001). However, the number of GABA-positive neurons in the 12-month-old CUMS-E4 group was significantly less than that of the age-matched CUMS-E3 and control groups (for the CUMS-E3 group, 82.83 ± 4.46 vs. 158.3 ± 8.38, *p* < 0.001; for the control-E4 group, 82.83 ± 4.46 vs. 192.3 ± 5.41, *p* < 0.001; for the control-E3 group, 82.83 ± 4.46 vs. 194.2 ± 3.73, *p* < 0.001). Consistent with the staining results of the prefrontal cortex, the number of GABA-positive neurons in the hippocampal dentate gyrus (DG) region for the 12-month-old CUMS-E4 group was significantly lower than that of the age-matched CUMS-E3 and control groups (for the CUMS-E3 group, 27.83 ± 3.24 vs. 45.33 ± 2.04, *p* < 0.001; for the control-E4 group, 27.83 ± 3.24 vs. 54.38 ± 3.38, *p* < 0.001; for the control-E3 group, 27.83 ± 3.24 vs. 54.83 ± 2.34, *p* < 0.001) (Fig. 5). Therefore, our data suggest that the number of GABAergic neurons in ApoE4-TR mice is irreversibly reduced following early-life CUMS intervention.

**Fig. 6:**
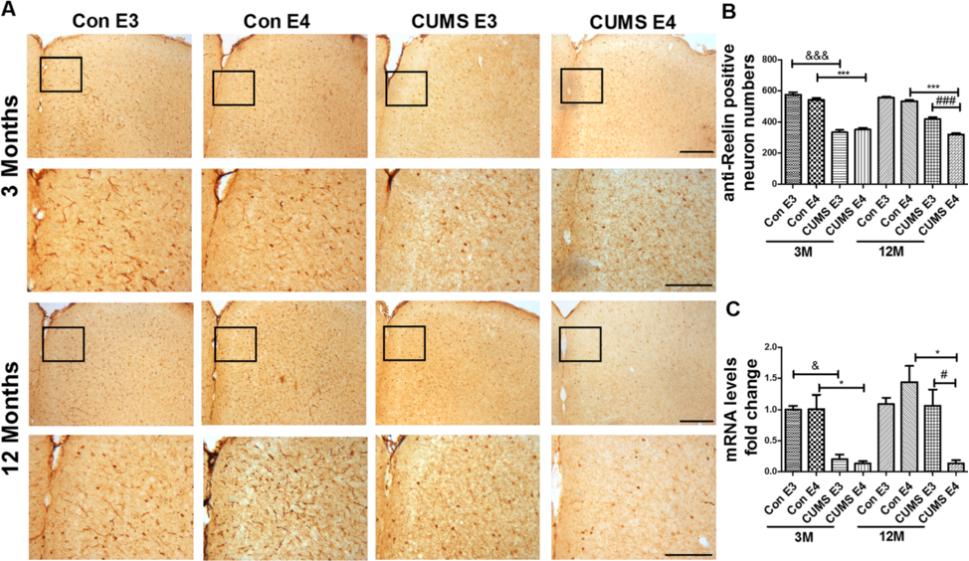
Decreased expression of Reelin in the prefrontal cortex of 12-month-old ApoE4-TR mice that underwent early-life CUMS intervention. **a** Anti-Reelin immunohistochemical staining in the prefrontal cortex of ApoE-TR mice at 3 months and 12 months. The anti-Reelin-positive neurons were shown as dark brown dots or claws. **b** The quantified positively-stained Reelin in the prefrontal cortex. *N* = 5 per group, expressed as mean ± SEM. &&& *p* < 0.001, ### *p* < 0.001, ***p < 0.001. Scale bars are 200 um. **c** The expression of Reelin mRNA analyzed by real time RT-PCR in the prefrontal cortex. *N* = 3 per group, expressed as mean ± SEM. one-factor ANOVA of statistical methods was used. & *p* < 0.05, # *p* < 0.05, *p < 0.05

**Fig. 7:**
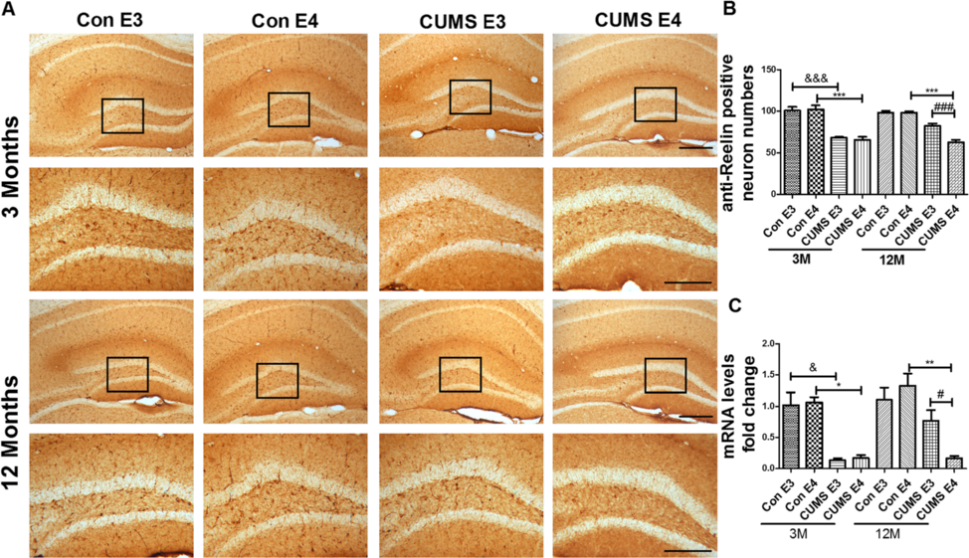
Decreased expression of Reelin in the hippocampus of 12-month-old ApoE4-TR mice that underwent early-life CUMS intervention. **a** Anti-Reelin immunohistochemical staining in the DG region of the hippocampus of 3-month-old and 12-month-old ApoE-TR mice. The anti-Reelin-positive neurons were shown as dark brown dots or claws. **b** The quantified positively-stained Reelin in the DG region of the hippocampus. *N* = 6 per group, expressed as mean ± SEM. &&& *p* < 0.001, ### *p* < 0.001, ***p < 0.001. Scale bars are 200 um. **c** The expression of Reelin mRNA analyzed by real time RT-PCR in the hippocampus. *n* = 3 per group, expressed as mean ± SEM. one-factor ANOVA of statistical methods was used. & *p* < 0.05, # *p* < 0.05, *p < 0.05, **p < 0.01

The expression of Reelin in the 3-month-old CUMS-E3 group and CUMS-E4 group was greatly reduced when compared with that of the age-matched control groups either in the prefrontal cortex (Fig. 6) (for ApoE3, 332.0 ± 15.29 vs. 574.2 ± 17.02, *p* < 0.001; for ApoE4, 350.8 ± 9.71 vs. 540.8 ± 12.23, *p* < 0.001) or in the DG region of the hippocampus (Fig. 7) (for ApoE3, 67.95 ± 1.18 vs. 100.8 ± 4.36, *p* < 0.001; for ApoE4, 65.65 ± 3.60 vs. 102.3 ± 5.03, *p* < 0.001). Compared with other groups, Reelin expression at the 12^th^ month for the CUMS-E4 group was decreased in both the prefrontal cortex (for the CUMS-E3 group, 318.2 ± 9.38 vs. 417.6 ± 12.50, *p* < 0.001; for the control-E4 group, 318.2 ± 9.38 vs. 534.0 ± 7.53, *p* < 0.001; for the control-E3 group, 318.2 ± 9.38 vs. 556.6 ± 6.39, *p* < 0.001) and the DG of the hippocampus (for the CUMS-E3 group, 62.45 ± 2.92 vs. 82.25 ± 2.95, *p* < 0.001; for the control-E4 group, 62.45 ± 2.92 vs. 98.20 ± 1.59, *p* < 0.001; for the control-E3 group, 62.45 ± 2.92 vs. 98.00 ± 2.28, *p* < 0.001). At 3 months old, the mRNA level of Reelin in the CUMS groups was markedly less that of the control groups either in the prefrontal cortex (*p* < 0.05) (Fig. 6c) or in the hippocampus (*p* < 0.05) (Fig. 7c). At 12 months, only the CUMS-E4 group exhibited a decreased Reelin mRNA level, either in the prefrontal cortex (for the CUMS-E3 group, 0.134 ± 0.052 vs. 1.063 ± 0.256, *p* < 0.05) or in the hippocampus (for the CUMS-E3 group, 0.165 ± 0.0365 vs. 0.766 ± 0.175, *p* < 0.05). These results suggest that the expression of Reelin is irreversibly diminished in the ApoE4-TR mice following early-life CUMS intervention.

**Fig. 8:**
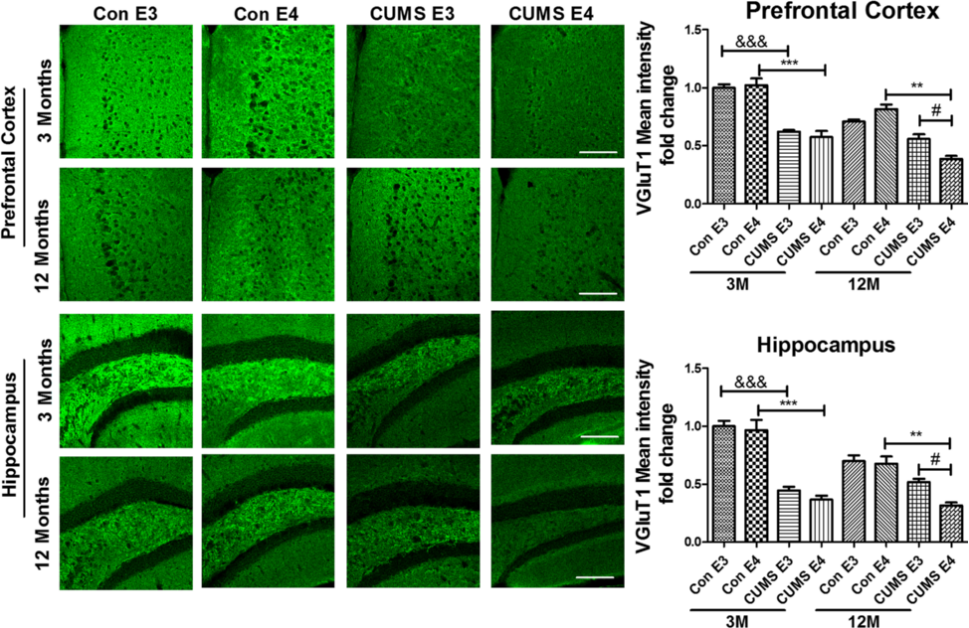
Decreased the levels of Glutamatergic neurons in the prefrontal cortex and the DG of the hippocampus in the 12-month-old ApoE4-TR mice that underwent early-life CUMS intervention. Immunofluorescent staining of the presynaptic gutamatergic transporter VGluT1 in the prefrontal cortex and the DG of the hippocampus in the ApoE-TR mice. Representative images (X20 magnification) are presented on the left. Quantification of the results (the mean intensity) was performed by computerized image analysis is shown on the right. *N* = 6 per group, expressed as mean ± SEM., one-factor ANOVA was adopt to analysis the data. &&& *p* < 0.001, # *p* < 0.05, *** *p* < 0.001, ** *p* < 0.01. Scale bars are 100 um

Due to the excitatory and inhibitory balance of neuronal network activity is essential for normal brain function and may be of particular importance to memory [39]. The immunofluorescent staining of the presynaptic glutamatergic vesicular transporter (VGluT1), one of the significant Glutamatergic neurons marker, was detected in the prefrontal cortex and the DG of the hippocampus. As shown in Fig. 8 (with green positive staining), the mean fluorescence intensity of VGluT1 in the prefrontal cortex of 3-month-old CUMS-E3 and age-matched CUMS-E4 group was less than that of the age-matched control groups. However, the mean fluorescence intensity of VGluT1 in the 12-month-old CUMS-E4 group was significantly less than that of the age-matched CUMS-E3 and control groups. Consistent with the staining results of the prefrontal cortex, the mean fluorescence intensity of VGluT1 in the DG of the hippocampus for the 12-month-old CUMS-E4 group was significantly lower than that of the age-matched CUMS-E3 and control groups. Therefore, our data suggest that the number of Glutamatergic neuron in ApoE4-TR mice is irreversibly reduced following early-life CUMS intervention.

### Reelin-Fyn-NMDAR2B signaling pathway activity is decreased in the prefrontal cortex and hippocampus of 12-month-old ApoE4-TR mice that underwent early-life CUMS intervention

**Fig. 9:**
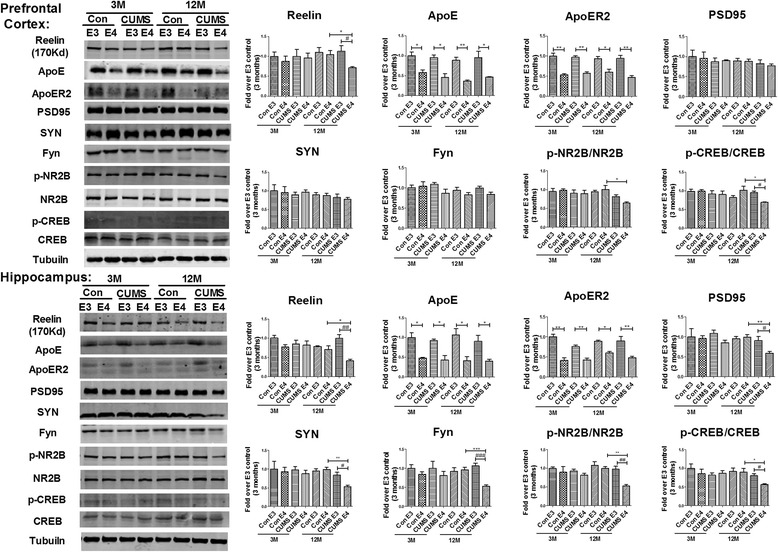
Decreased expression of Reelin-Fyn-NMDAR2B in the prefrontal cortex and hippocampus of 12-month-old ApoE4-TR mice that underwent early-life CUMS intervention. The levels of Reelin (170Kd), ApoE, ApoER2, PSD95, SYN, Fyn, p-NR2B, NR2B, p-CREB and CREB were detected respectively by Western blot in the prefrontal cortex and hippocampus. *N* = 6 per group, expressed as mean ± SEM. one-factor ANOVA was used to analysis the data. *p < 0.05, **p < 0.01, ***p < 0.001, # *p* < 0.05, ## *p* < 0.01, ### *p* < 0.001

Given the Reelin-ApoER2-Fyn-NMDA signaling pathway plays an important role in learning and memory [40], we examined the expression of Reelin, ApoE, ApoER2, Fyn, PSD95, SYN, NR2A, and phosphorylated NR2B and phosphorylated-CREB in the prefrontal cortex and hippocampus by Western-blotting. In the prefrontal cortex and hippocampus, the level of ApoE and ApoER2 in the ApoE4 control and stress groups was significantly lower than that of the ApoE3 groups (*p* < 0.05 and *p* < 0.01, respectively) (Fig. 9). At the age of 12 months, the level of Reelin (170Kda) in the CUMS-E4 group was significantly lower compared with that of the CUMS-E3 group (41.77 % ±3.11 % decline, *p* < 0.05 for prefrontal-cortex and 57.83 % ±4.27 % decline, *p* < 0.01 for hippocampus) and of the control-E4 group (33.53 % ±3.10 % decline, *p* < 0.05 for prefrontal-cortex and 29.44 % ±4.27 % decline, *p* < 0.05 for hippocampus). The level of PSD95, SYN and Fyn in the hippocampus of the CUMS-E4 group was lower when compared to the CUMS-E3 group (for PSD95, 31.77 % ±4.24 % decline, *p* < 0.05; for Syn, 30.50 % ±3.92 % decline, *p* < 0.05; for Fyn, 54.22 % ±4.71 % decline, *p* < 0.001) and the control-E4 group (for PSD95, 40.20 % ±4.24 % decline, *p* < 0.01; for Syn, 45.60 % ±3.92 % decline, *p* < 0.01; for Fyn, 43.67 % ±4.71 % decline, *p* < 0.001). The expression of PSD95, SYN and Fyn in the prefrontal cortex of the CUMS-E4 group were also diminished when compared with those of the CUMS-E3 and Control-E4 group, although the difference was not significant (*p* > 0.05). The level of p-NR2B in the CUMS-E4 group was less than that of the CUMS-E3 group (16.97 % ±3.32 % decline, *p* > 0.05 for prefrontal-cortex and 44.67 % ±3.85 % decline, *p* < 0.01 for hippocampus) and of the Control-E4 group (36.25 % ±3.32 % decline, *p* < 0.05 for prefrontal-cortex and 46.85 % ±3.84 % decline, *p* < 0.01 for hippocampus). However, the expression of NMDAR2B, either in the prefrontal cortex or in the hippocampus, was not significantly different among all groups. We also checked the levels of phosphorylated CREB and total CREB, which are the downstream proteins of the Reelin-Fyn-NR2B signaling pathway. The level of p-CREB in the 12 M CUMS-E4 group was less than that of the CUMS-E3 group (26.05 % ±0.97 % decline, *p* < 0.05 for prefrontal-cortex and 24.02 % ±2.52 % decline, *p* < 0.05 for hippocampus) and of the Control-E4 group (28.60 % ±0.97 % decline, *p* < 0.05 for prefrontal-cortex and 32.89 % ±2.52 % decline, *p* < 0.05 for hippocampus). However, the expression of total CREB, either in the prefrontal cortex or in the hippocampus, was not significantly different among all groups. These results indicate the activity of the Reelin-ApoER2-Fyn-NMDAR2B pathway is decreased in the 12-month-old CUMS-E4 mice.

**Fig. 10:**
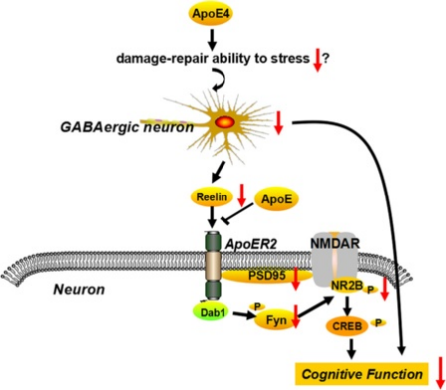
The cognitive impairment mediated by the Reelin-Fyn-NR2B signaling pathway in 12-month-old ApoE4-TR mice that underwent early-life early CUMS intervention. ApoE secreted by astrocytes and Reelin produced by GABAergic neurons compete to bind to ApoER2 in neurons. Reelin binding to ApoER2 sequentially induces the activation of Dab1, SRC family tyrosine kinases (Fyn) and NMDAR2B subunits, which in turn promote the influx of Ca2+ and expression of the cognition-associated proteins. Following early-life CUMS intervention, the number of GABAergic neurons and the level of Reelin in the prefrontal cortex and the DG of the hippocampus of the 12-month-old ApoE4-TR mice, compared with those of other groups, are reduced, thus down-regulating the activation of Fyn/p-NR2B

## Discussion

In the present study, we created a depression model by treating 3-month-old ApoE-TR mice with chronic unpredictable mild stress (CUMS). After the CUMS intervention, the mice were able to recover from depression by the age of 12 months. However, 12-month-old CUMS-E4 mice displayed a decline in spatial cognitive abilities when compared with CUMS-E3 mice of the same age. Interestingly, we found the number of GABAergic neurons and Glutamatergic neurons and the expression of Reelin in the prefrontal cortex and hippocampus were reduced in CUMS-E4 mice. Additionally, the expression of Reelin and Fyn and the phosphorylation of NMDAR2B and CREB, all of which are critical to proper maintenance of cognitive pathways, were also decreased in the CUMS-E4 mice.

Our study demonstrates 3-month-old ApoE-TR mice, having undergone CUMS intervention, performed worse in the Morris maze test than the control groups (Fig. 2i, Fig. 2k-m), and their exercise capacity (swimming speed) also decreased (Fig. 2j). These results may represent a manifestation of depression symptoms or real cognitive impairment. When the mice were allowed to recover after the CUMS intervention, their depression-like behavior disappeared (Fig. 3a-f) and exercise capacity was restored (Fig. 3h) by the age of 12 months. The CUMS-E3 group’s scores on the Morris maze test were not significantly different from those of the control group, suggesting that the decline in performance and decreased motor ability of the young ApoE-TR mice in the Morris water maze test represented a pseudo-dementia, resulting from the CUMS intervention. These findings are consistent with clinical studies of depression patients who often manifest symptoms of pseudo-dementia [41, 42].

Recent studies [43, 44] have reported that it takes the naturally-aging ApoE4-TR mice 14–16 months to display cognitive decline. In the current study, the scores of 12-month-old CUMS-E4 mice in the Morris maze test (including escape latency, target quadrant time and number of crossings, as shown in Fig. 3g and Fig. 3i-k) were significantly lower than those of the control groups and CUMS-E3 mice, suggesting that early-life CUMS intervention accelerates the decline of spatial learning and memory of ApoE4-TR mice.

Our findings are consistent with the clinical observation that early-life depression in ApoE4 carriers leads to impaired cognitive function in older age. Clinical studies have shown that a history of depressive symptoms [45] and the *APOE4* genotype [3] are independent contributors to cognitive decline (CD). Moreover, the influence of depression on CD is greater in individuals with the *APOE4* genotype; depression and the *APOE4* genotype may act concomitantly to affect an individual’s cognitive reserve capacity in old age [46].

Consistent with clinical autopsy of patients with depression [17, 47, 48] and depression animal models [37], which reveal a great loss of GABAergic neurons in the brain, we found that the GABAergic neurons in young ApoE-TR mice were greatly reduced following CUMS intervention. Of note, the number of GABAergic neurons in CUMS-E3 mice could be partially restored by 12 months of age; however, the number of GABAergic neurons in the hippocampus of 12-month-old CUMS-E4 mice was significantly less than that of CUMS-E3 mice. We also found that the number of the Glutamatergic neurons shows paralleled with the change of GABAergic neurons in the prefrontal cortex and the DG region of the hippocampus, some studies have reported that the social defeat depressive model mice have appeared the dysfunctional activities of GABAergic and Glutamatergic neurons in the prefrontal cortex [39]. These results further confirmed the early intervention has a greatly impact in the ApoE4-TR mice. The level of ApoE in the prefrontal cortex and hippocampus of the ApoE4 groups was significantly lower than that of the ApoE3 groups, so we speculate that after the CUMS treatment, the reduced ability of ApoE4-TR mice to repair GABAergic neurons may be associated with lower ApoE levels in the brain. Such findings are consistent with studies reporting that compared to ApoE3, ApoE4 possesses weaker damage-repair ability to fight against acute inflammatory reaction and apoptosis [49]. Huang et al. reported [50–53] that female apoE4-TR mice had an age-dependent decline in hilar GABAergic interneurons that correlated with the extent of learning and memory deficits in aged mice, but not in the male apoE4-TR mice. Meanwhile, inhibitory interneuron progenitor transplantation or GABAA receptor potentiator pentobarbital (PB) restores normal learning and memory in ApoE4-TR mice [54, 55]. Huang’s research illustrated that decline in GABA signaling play an important role in the cognitive dysfunction in ApoE4-TR mice, which is consistent with our results. In order to remove the effect of estrogen and estrous cycle on the emotional and cognitive processes in the female mice, only the male apoE4-TR mice were used in our study.

Given GABAergic neurons secrete Reelin [56], in the prefrontal cortex and hippocampus [18], we sought to explore the relationship between the two. Our immunohistochemical results showed that the decrease in positively-stained Reelin ran parallel with that of the GABAergic neurons in both the prefrontal cortex and the DG of the hippocampus. Reelin is an extracellular matrix protein with molecular weight varying from 410Kda, 330Kda, 170Ka or lower. In our study, only the band of 170Kda Reelin were detected by western-blot, however, all fragments were detected by immunohistochemical staining and qPCR. In 3-month-old CUMS ApoE3/4 mice, the Reelin-positive stained and mRNA of Reelin were significantly lower than that in the control, there was no obviously difference in the protein of Reelin (170 kDa) detected by western-blot. Of note, we found that the full Reelin (immunohistochemical and qPCR) and 170 kDa Reelin (western blot) of middle-aged ApoE4 mice were significant reduced in the brain. Taken together, these findings suggest that the reduction of GABAergic neurons decreases the synthesis of Reelin, rather than increasing its degradation, in the 12-month-old CUMS-E4 group.

Although ApoE and Reelin competitively bind to ApoER2 on the cell membrane [19, 40], our research indicates that the expression of ApoE and ApoER2 remains unaffected by stress and age-related changes. In the brain, the binding of Reelin to ApoER2 activates the SRC family tyrosine kinases (Fyn) and leads to the phosphorylation of NR2B, resulting in an increased calcium influx and activation of downstream signaling pathways associated with cognitive function [20, 40]. Our results reveal that in the hippocampus of 12-month-old CUMS ApoE4-TR mice, levels of Fyn, Syn, PSD95, phosphorylated NR2B and phosphorylated CREB were significantly lower than those of other groups. Our previous studies have found the level of NR2B in E4FAD mice (generated by crossing 5xFAD mice and h-APOE4-TR mice) were significantly decreased in the hippocampus [57]. Therefore, we speculate that decreased expression of Reelin may lead to lowered activation of downstream Fyn and declined phosphorylation of NR2B.

## Conclusions

Herein the current study demonstrates that early life stress leads to spatial learning and memory decline in middle-aged ApoE4-TR mice. CUMS intervention also causes a loss of GABAergic neurons and a reduced synthesis of Reelin in the prefrontal cortex and hippocampus that is evident in middle-aged ApoE4-TR mice. The decreased level of Reelin impairs the activation of the Fyn-NMDAR2B-CREB signaling pathway, which leads to the cognitive impairment of ApoE4-TR mice (Fig. 10). This study further confirms the clinical relationship among ApoE4, depression and cognitive impairment and provides insight regarding the mechanisms linking early life stress to cognitive impairment in middle-aged ApoE4-TR mice. Future studies are needed to investigate the mechanism(s) through which early-life stress induces a sustained reduction of GABAergic neurons in ApoE4-TR mice.

## Abbreviations

AD: Alzheimer’s disease
apoE: apolipoprotein E
ApoER2: apolipoprotein E receptors2
CD: cognitive decline
CREB: cyclic AMP response element binding protein
CUMS: chronic unpredictable mild stress
CUMS-E3: CUMS-treated ApoE3
CUMS-E4: CUMS-treated ApoE4
DG: dentate gyrus
EFAD mice: Tg-mice expressing 5xFAD mutations and human APOE2, APOE3, or APOE4
MCI: mild cognitive impairment
NMDAR: N-methyl-D-aspartate receptor
OFT: open field test
p: Phosphorylated
PB: potentiator pentobarbital
p-CREB: phosphorylated cAMP-response element binding protein
PSD95: postsynaptic density protein 95
ROI: regions of interest
SFKs: SRC family kinases
SYN: synaptophysin
TR: target replacement
TST: tail suspension test
VLDLR: very-low-density lipoprotein receptor

## References

[1] Mahley RW, Weisgraber KH, Huang Y (2006). Apolipoprotein E4: a causative factor and therapeutic target in neuropathology, including Alzheimer’s disease. Proc Natl Acad Sci U S A.

[2] Huang Y, Liu XQ, Wyss-Coray T, Brecht WJ, Sanan DA, Mahley RW (2001). Apolipoprotein E fragments present in Alzheimer’s disease brains induce neurofibrillary tangle-like intracellular inclusions in neurons. Proc Natl Acad Sci U S A.

[3] Huang Y (2006). Apolipoprotein E, and Alzheimer disease. Neurology.

[4] Farrer LA, Cupples LA, Haines JL, Hyman B, Kukull WA, Mayeux R (1997). Effects of age, sex, and ethnicity on the association between apolipoprotein E genotype and Alzheimer disease. A meta-analysis. APOE and Alzheimer Disease Meta Analysis Consortium. JAMA.

[5] Corder EH, Saunders AM, Strittmatter WJ, Schmechel DE, Gaskell PC, Small GW (1993). Gene dose of apolipoprotein E type 4 allele and the risk of Alzheimer’s disease in late onset families. Science.

[6] Saunders AM, Strittmatter WJ, Schmechel D, George-Hyslop PH, Pericak-Vance MA, Joo SH (1993). Association of apolipoprotein E allele epsilon 4 with late-onset familial and sporadic Alzheimer’s disease. Neurology.

[7] Stewart R, Russ C, Richards M, Brayne C, Lovestone S, Mann A (2001). Depression, APOE genotype and subjective memory impairment: a cross-sectional study in an African-Caribbean population. Psychol Med.

[8] Rapp MA, Schnaider-Beeri M, Grossman HT, Sano M, Perl DP, Purohit DP (2006). Increased hippocampal plaques and tangles in patients with Alzheimer disease with a lifetime history of major depression. Arch Gen Psychiatry.

[9] Jorm AF, van Duijn CM, Chandra V, Fratiglioni L, Graves AB, Heyman A (1991). Psychiatric history and related exposures as risk factors for Alzheimer’s disease: a collaborative re-analysis of case-control studies. EURODEM Risk Factors Research Group. Int J Epidemiol.

[10] Kokmen E, Beard CM, Chandra V, Offord KP, Schoenberg BS, Ballard DJ (1991). Clinical risk factors for Alzheimer’s disease: a population-based case-control study. Neurology.

[11] Park S, Nam YY, Sim Y, Hong JP (2015). Interactions between the apolipoprotein E epsilon4 allele status and adverse childhood experiences on depressive symptoms in older adults. Eur J Psychotraumatol.

[12] Kim DH, Payne ME, Levy RM, MacFall JR, Steffens DC (2002). APOE genotype and hippocampal volume change in geriatric depression. Biol Psychiatry.

[13] Steffens DC, Plassman BL, Helms MJ, Welsh-Bohmer KA, Saunders AM, Breitner JC (1997). A twin study of late-onset depression and apolipoprotein E epsilon 4 as risk factors for Alzheimer’s disease. Biol Psychiatry.

[14] Geda YE, Knopman DS, Mrazek DA, Jicha GA, Smith GE, Negash S (2006). Depression, apolipoprotein E genotype, and the incidence of mild cognitive impairment: a prospective cohort study. Arch Neurol.

[15] Kim JM, Stewart R, Kim SY, Kim SW, Bae KY, Yang SJ (2011). Synergistic associations of depression and apolipoprotein E genotype with incidence of dementia. Int J Geriatr Psychiatry.

[16] Rajan KB, Wilson RS, Skarupski KA, de Leon CF M, Evans DA (2014). Gene-behavior interaction of depressive symptoms and the apolipoprotein E {varepsilon}4 allele on cognitive decline. Psychosom Med.

[17] Rajkowska G, O’Dwyer G, Teleki Z, Stockmeier CA, Miguel-Hidalgo JJ (2007). GABAergic neurons immunoreactive for calcium binding proteins are reduced in the prefrontal cortex in major depression. Neuropsychopharmacology.

[18] Alcantara S, Ruiz M, D’Arcangelo G, Ezan F, de Lecea L, Curran T (1998). Regional and cellular patterns of reelin mRNA expression in the forebrain of the developing and adult mouse. J Neurosci.

[19] D’Arcangelo G, Homayouni R, Keshvara L, Rice DS, Sheldon M, Curran T (1999). Reelin is a ligand for lipoprotein receptors. Neuron.

[20] Herz J, Chen Y (2006). Reelin, lipoprotein receptors and synaptic plasticity. Nat Rev Neurosci.

[21] Niu S, Renfro A, Quattrocchi CC, Sheldon M, D’Arcangelo G (2004). Reelin promotes hippocampal dendrite development through the VLDLR/ApoER2-Dab1 pathway. Neuron.

[22] Niu S, Yabut O, D’Arcangelo G (2008). The Reelin signaling pathway promotes dendritic spine development in hippocampal neurons. J Neurosci.

[23] Fatemi SH (2011). Reelin, a marker of stress resilience in depression and psychosis. Neuropsychopharmacology.

[24] Impagnatiello F, Guidotti AR, Pesold C, Dwivedi Y, Caruncho H, Pisu MG (1998). A decrease of reelin expression as a putative vulnerability factor in schizophrenia. Proc Natl Acad Sci U S A.

[25] Teixeira CM, Martin ED, Sahun I, Masachs N, Pujadas L, Corvelo A (2011). Overexpression of Reelin prevents the manifestation of behavioral phenotypes related to schizophrenia and bipolar disorder. Neuropsychopharmacology.

[26] Willner P, Muscat R, Papp M (1992). Chronic mild stress-induced anhedonia: a realistic animal model of depression. Neurosci Biobehav Rev.

[27] Knouff C, Hinsdale ME, Mezdour H, Altenburg MK, Watanabe M, Quarfordt SH (1999). Apo E structure determines VLDL clearance and atherosclerosis risk in mice. J Clin Invest.

[28] Moreau JL, Scherschlicht R, Jenck F, Martin JR (1995). Chronic mild stress-induced anhedonia model of depression; sleep abnormalities and curative effects of electroshock treatment. Behav Pharmacol.

[29] Forbes NF, Stewart CA, Matthews K, Reid IC (1996). Chronic mild stress and sucrose consumption: validity as a model of depression. Physiol Behav.

[30] Pothion S, Bizot JC, Trovero F, Belzung C (2004). Strain differences in sucrose preference and in the consequences of unpredictable chronic mild stress. Behav Brain Res.

[31] Pellow S, Chopin P, File SE, Briley M (1985). Validation of open:closed arm entries in an elevated plus-maze as a measure of anxiety in the rat. J Neurosci Methods.

[32] Rodgers RJ, Cole JC, Aboualfa K, Stephenson LH (1995). Ethopharmacological analysis of the effects of putative ‘anxiogenic’ agents in the mouse elevated plus-maze. Pharmacol Biochem Behav.

[33] Heimrich B, Claus H, Schwegler H, Haas HL (1989). Hippocampal mossy fiber distribution and long-term potentiation in two inbred mouse strains. Brain Res.

[34] Crowley JJ, Jones MD, O’Leary OF, Lucki I (2004). Automated tests for measuring the effects of antidepressants in mice. Pharmacol Biochem Behav.

[35] Morris R (1984). Developments of a water-maze procedure for studying spatial learning in the rat. J Neurosci Methods.

[36] Vorhees CV, Williams MT (2006). Morris water maze: procedures for assessing spatial and related forms of learning and memory. Nat Protoc.

[37] Kalueff AV, Nutt DJ (2007). Role of GABA in anxiety and depression. Depress Anxiety.

[38] Campo CG, Sinagra M, Verrier D, Manzoni OJ, Chavis P (2009). Reelin secreted by GABAergic neurons regulates glutamate receptor homeostasis. PLoS One.

[39] Veeraiah P, Noronha JM, Maitra S, Bagga P, Khandelwal N, Chakravarty S (2014). Dysfunctional glutamatergic and gamma-aminobutyric acidergic activities in prefrontal cortex of mice in social defeat model of depression. Biol Psychiatry.

[40] D’Arcangelo G (2005). Apoer2: a reelin receptor to remember. Neuron.

[41] Gucuyener DO, Yenilmez C, Ayranci U, Ozdemir F, Uzuner N, Ozkan S (2010). An analysis of changes in cerebral blood flood velocities in depressive pseudo-dementia and Alzheimer disease patients. Neurologist.

[42] Banga A, Gyurmey T, Matuskey D, Connor DF, Kaplan RF, Steffens DC (2013). Late-life onset bipolar disorder presenting as a case of pseudo-dementia: a case discussion and review of literature. Yale J Biol Med.

[43] Siegel JA, Haley GE, Raber J (2012). Apolipoprotein E isoform-dependent effects on anxiety and cognition in female TR mice. Neurobiol Aging.

[44] Rijpma A, Jansen D, Arnoldussen IAC, Fang XT, Wiesmann M, Mutsaers MPC (2013). Sex differences in presynaptic density and neurogenesis in middle-aged ApoE4 and ApoE knockout mice. J Neurodegener Dis.

[45] Sachs-Ericsson N, Joiner T, Plant EA, Blazer DG (2005). The influence of depression on cognitive decline in community-dwelling elderly persons. Am J Geriatr Psychiatry.

[46] Corsentino EA, Sawyer K, Sachs-Ericsson N, Blazer DG (2009). Depressive symptoms moderate the influence of the apolipoproteine epsilon4 allele on cognitive decline in a sample of community dwelling older adults. Am J Geriatr Psychiatry.

[47] Benes FM, Berretta S (2001). GABAergic interneurons: implications for understanding schizophrenia and bipolar disorder. Neuropsychopharmacology.

[48] Guidotti A, Auta J, Davis JM, Di-Giorgi-Gerevini V, Dwivedi Y, Grayson DR (2000). Decrease in reelin and glutamic acid decarboxylase67 (GAD67) expression in schizophrenia and bipolar disorder: a postmortem brain study. Arch Gen Psychiatry.

[49] McColl BW, McGregor AL, Wong A, Harris JD, Amalfitano A, Magnoni S (2007). APOE epsilon3 gene transfer attenuates brain damage after experimental stroke. J Cereb Blood Flow Metab.

[50] Knoferle J, Yoon SY, Walker D, Leung L, Gillespie AK, Tong LM (2014). Apolipoprotein E4 produced in GABAergic interneurons causes learning and memory deficits in mice. J Neurosci.

[51] Estus S, Leung L, Andrews-Zwilling Y, Yoon SY, Jain S, Ring K (2012). Apolipoprotein E4 causes age- and sex-dependent impairments of Hilar GABAergic interneurons and learning and memory deficits in mice. PLoS One.

[52] Andrews-Zwilling Y, Bien-Ly N, Xu Q, Li G, Bernardo A, Yoon SY (2010). Apolipoprotein E4 causes age- and Tau-dependent impairment of GABAergic interneurons, leading to learning and memory deficits in mice. J Neurosci.

[53] Li G, Bien-Ly N, Andrews-Zwilling Y, Xu Q, Bernardo A, Ring K (2009). GABAergic interneuron dysfunction impairs hippocampal neurogenesis in adult apolipoprotein E4 knockin mice. Cell Stem Cell.

[54] Tong LM, Yoon SY, Andrews-Zwilling Y, Yang A, Lin V, Lei H (2016). Enhancing GABA signaling during middle adulthood prevents age-dependent GABAergic interneuron decline and learning and memory deficits in ApoE4 mice. J Neurosci.

[55] Tong LM, Djukic B, Arnold C, Gillespie AK, Yoon SY, Wang MM (2014). Inhibitory interneuron progenitor transplantation restores normal learning and memory in ApoE4 knock-in mice without or with Abeta accumulation. J Neurosci.

[56] Fatemi SH (2005). Reelin glycoprotein: structure, biology and roles in health and disease. Mol Psychiatry.

[57] Liu DS, Pan XD, Zhang J, Shen H, Collins NC, Cole AM (2015). APOE4 enhances age-dependent decline in cognitive function by down-regulating an NMDA receptor pathway in EFAD-Tg mice. Mol Neurodegener.

